# Nicotine Pouch Advertising and Sales in Australia: A Qualitative Study of Younger People's Experiences

**DOI:** 10.1002/hpja.70239

**Published:** 2026-08-23

**Authors:** Michelle I. Jongenelis, Runze Li

**Affiliations:** ^1^ Melbourne Centre for Behaviour Change, Melbourne School of Psychological Sciences The University of Melbourne Parkville Australia

**Keywords:** advertising, nicotine pouches, social influencers, tobacco industry

## Abstract

**Issue Addressed:**

Their position as a target demographic of the tobacco industry means younger people are best placed to provide information on the activities of industry actors in relation to nicotine pouches. Accordingly, we aimed to qualitatively examine younger people's exposure to pouch advertising, from where pouches are being sourced, and any notable retailer practices and industry innovations.

**Methods:**

Australians aged 16–39 years were recruited to participate in one of 12 focus groups held February to April 2025. Groups ranged in size from 6 to 10 participants, with a total of 98 participants in attendance. Data were synthesised using template analysis.

**Results:**

Almost all groups reported being exposed to nicotine pouch advertising, despite such advertising being illegal in Australia. Social media was the most common source of advertising exposure, with TikTok the most frequently cited platform. Participants reported that advertising typically featured attractive influencers portraying pouches as “cool” and describing nicotine as “natural”. Tobacconists and convenience stores were key product sources. Few retailer practices were identified, with these broadly relating to product cost and inducements.

**Conclusions:**

Results provide initial insights into the insidiousness of the marketing strategies being adopted to sell nicotine pouches to younger people.

**So What?:**

Findings highlight an urgent need for action that protects younger people from tobacco companies and the influencers they engage to promote their addictive products. Action should include robust regulation of the online environment and holding social media platforms responsible for their failure to enforce policies prohibiting product advertising and sales.

## 1 Introduction

As smoking rates continue to decline globally, tobacco companies are increasingly targeting young people to maintain profitability [1, 2, 3]. This has led to the emergence of a variety of nicotine products, the most recent of which include nicotine pouches: small, permeable bags filled with nicotine and other chemicals that are placed between the lip and the gum where the contents dissolve into the bloodstream [4].

International evidence indicates that use of nicotine pouches has increased rapidly in recent years, particularly among men, young adults, and those who report smoking and/or vaping [5, 6]. In Australia, the context of the present study, there remains limited information about prevalence rates as national monitoring frameworks have yet to report on pouch use in detail. Early insights from the 2025 National Drug Strategy Household Survey indicate that 1.8% of Australians aged 14+ years reported pouch use in the preceding 12 months, with use among 14‐ to 17‐year‐olds at 1.2% and use among 18‐ to 24‐year‐olds at 8.4% [7]. Further evidence from surveys conducted with 16‐ to 39‐year‐olds and focus groups conducted with adolescents aged 14–17 years and young adults aged 18–24 years suggests some have used the products and many may be susceptible to initiation [8, 9, 10]. This is despite regulations prohibiting the sale and advertising of nicotine pouches in Australia [11].

Given the global nature of social media, it is likely that younger Australians are being exposed to industry‐funded content promoting nicotine pouch use despite the existing prohibition on advertising. For example, prior research analysing the portrayal of ZYN (a nicotine pouch brand produced by Swedish Match, a subsidiary of Philip Morris International) on TikTok found evidence of content creators with commercial links to nicotine pouch manufacturers actively selling pouches on the platform [12]. This analysis also found that the content being produced was targeted towards young males and overwhelmingly positive in sentiment, with nicotine pouches portrayed as fun products. Humour was often employed, and creators typically omitted or minimised the risk of addiction associated with the products. Prior qualitative research exploring young people's views of nicotine pouches in the UK also identified the use of social media to promote nicotine pouches [13]. This research additionally found a link between the marketing of nicotine pouches and sports, with males reporting on Velo pouches (produced by British American Tobacco) being promoted on McLaren Formula 1 cars and by footballers and football managers. Consistent with the findings observed in the TikTok content analysis described above, participants held the view that the marketing of pouches was aimed more at males than females.

Research exploring nicotine pouch advertising in Australia is lacking. Given younger people are a target demographic of the tobacco and nicotine industry, they are best placed to provide information on the activities of industry actors in relation to nicotine pouches. Accordingly, we sought to qualitatively examine younger people's exposure to nicotine pouch advertising. We also sought to explore younger people's perspectives on the sources of nicotine pouches, retailer practices, and industry innovations.

## 2 Method

### 2.1 Recruitment and Design

As part of a larger qualitative study exploring younger people's experiences with various nicotine products, Australians aged 16–39 years were recruited to participate in one of 12 focus groups conducted in Melbourne (Victoria) and Perth (Western Australia). Our decision to recruit those up to the age of 39 years reflects our desire to (1) capture Australians before they enter mid‐life and (2) recruit those who are the most prevalent users of vapes in Australia [14].

Two market research agencies (one based in Victoria and one in Western Australia) were commissioned to recruit participants. A purposive sampling approach was adopted, with the agencies instructed to recruit men and women within the eligible age range only. The recruitment agencies were also asked to ensure that approximately half of the participants in each focus group were currently vaping or had vaped in the past. This is because use of nicotine pouches has been found to be most common among those who vape [15, 16, 17].

Focus groups were conducted from February to April 2025. Groups were stratified by age (16 to 17‐year‐olds, 18 to 24‐year‐olds, and 25 to 39‐year‐olds) and gender (men, women) as individuals are more likely to feel comfortable sharing genuine perspectives in a homogenous group [18]. The specific age bands used to stratify groups were chosen to ensure consistency with national surveys (in particular, Australia's National Drug Strategy Household Survey) and prior quantitative work conducted on nicotine pouch use in Australia [9, 19].

### 2.2 Procedure

This study was approved by a university Human Research Ethics Committee and all participants provided written informed consent. A semi‐structured interview protocol comprising open‐ended questions was used to guide discussions. As part of the larger qualitative study, a range of topics relating to nicotine products was covered in 90‐min focus groups, with the present study focusing on the discussions held in relation to nicotine pouch advertising and sales.

To minimise priming, participants were first asked to describe what they know about nicotine pouches and any product‐related experiences. They were then provided with a definition of the product and images of example products were shown. Participants were subsequently asked more specific questions relating to the products (e.g., *What advertising for nicotine pouches have you come across? Where are these products being sourced?*). At the conclusion of the focus group, participants completed a short survey that collected demographic information and assessed use of various nicotine products. A gift card valued at AUD120 was given to all participants as reimbursement for their time and costs associated with group attendance.

### 2.3 Analysis

Audio‐recordings from the focus groups were transcribed verbatim and imported into NVivo for coding and analysis. Template analysis—a form of thematic analysis involving a priori development of a structured coding template that serves as a framework to map the analysis [20]—was used for data synthesis. Consistent with the aims of the study, parent nodes were created a priori for each of the following topics: (1) advertising exposure; (2) product source; (3) retailer practices; and (4) industry innovations. Child nodes were then created reflexively within each of these topics based on the specific content generated by participants. These nodes were subsequently reviewed and themes generated.

One researcher (MJ) coded all transcripts. To enhance the trustworthiness of the resulting interpretation, an independent researcher not involved in focus group delivery (RL) read the transcripts in their entirety and reviewed the identified themes. Discussions were then held between the primary coder and the independent researcher to refine the themes.

Differences by age and gender were explored using the Matrix Coding Query function available in NVivo.

## 3 Results

### 3.1 Sample Composition

Table 1 presents the final composition of each focus group. Groups ranged in size from 6 to 10 participants, with a total of 98 participants in attendance: *n* = 48 men, *n* = 49 women, and *n* = 1 non‐binary; *n* = 32 aged 16–17 years, *n* = 34 aged 18–24 years, and *n* = 32 aged 25–39 years. Ever use of nicotine pouches was reported by 16 participants.

**TABLE 1:** Focus group composition.

Gender	Age	Location	*N*	Pouch use (ever) *n*
Men	16–17 years	Victoria	6	0
		Western Australia	10	4
	18–24 years	Victoria	8	3
		Western Australia	8	3
	25–39 years	Victoria	6	2
		Western Australia	10	0
Women	16–17 years	Victoria	6	0
		Western Australia	10	2
	18–24 years	Victoria^a^	8	0
		Western Australia	10	0
	25–39 years	Victoria	6	1
		Western Australia	10	1

^a^ One participant in this group identified as non‐binary.

### 3.2 Advertising Exposure

Participants in almost all groups reported being exposed to nicotine pouch advertising. There was some variation by gender: all groups comprising men reported exposure compared to only half of the groups comprising women. Social media was overwhelmingly the most common source of advertising exposure, with TikTok the most frequently cited platform. Two distinct forms of advertising were described by participants: (1) paid, active promotion by social influencers, especially fitness/wellness influencers, and (2) seemingly organic and passive promotion by athletes (e.g., international soccer/football players) and podcasters, although some participants expressed scepticism that this promotion was truly organic. This is illustrated in the following quotes provided by men aged 25–39 years from Melbourne:

Interviewee 1: *I haven't really seen anyone personally use them, but they're everywhere. Like on every podcast I watch, on every TikTok or reels or something, people are always using them, all these Youtubers and stuff. Social media influencers*. *[unrelated discussion]*

Interviewee 2: *…Or there's a podcast where they're talking about ZYNs are so much better than vapes, and it feels better or whatever*.

Interviewee 3: *It feels like in these podcasts, a lot of these people are being paid by the ZYN company to mention it…*

Interviewee 4: *They're kind of just like*, “we'll be using them on the podcast” *or something. Maybe not shouting out the company*.

Interviewee 3: “I just gotta grab a ZYN”

Interviewee 4: *Yeah, stuff like that*.

Interviewer: *Okay. So, subtle advertising*.

Interviewee 4: *Yeah. Subtle advertising. Not having to say that they're being paid or anything to promote it because they're not technically promoting it. They just enjoy using it, right?*

Some participants provided specific information about the nature of the advertising and the messages being conveyed. These participants reported that nicotine pouches were promoted as being “*cool*”. Advertising also appeared to be aspirational, with participants reporting that they had seen pouches being advertised by “*really hot and cool*” influencers, and another commenting that those advertising the products are “*also the ones who have money to do a Europe summer*”. Some also reported on a trend they had observed on social media whereby fitness/wellness influencers describe nicotine as “*a natural product*” that is not “*bad*” in its own right, illustrated in the following quote provided by a woman aged 25–39 years from Perth:

Interviewee 1: *I know there's a lot currently going around TikTok and stuff like that, about the fact that nicotine is natural*. “It's not nicotine that's bad, it's what we're doing with it.”

Interviewer: *Right*

Interviewee 1: *Yes*, “it's okay, and look, now we can get nicotine in a tablet, and we can get it in this and it's okay because it's a natural product and it's not what we're doing with it”. *Again, I think it's already the next iteration. There's lots of people talking about that. When you start viewing those videos, we all know the algorithms do silly things and that's all you get*.

Two groups commented on nicotine pouches becoming a part of meme culture, illustrated by the following quote:

*Because there's a lot of, online there's a lot of memes about them that are very masculine* [Men, 18–24 years, Melbourne].

### 3.3 Product Source

Participants in almost all groups identified sources of nicotine pouches, with 16‐ to 17‐year‐olds and 18‐ to 24‐year‐olds more likely than 25‐ to 39‐year‐olds to do so. The most commonly reported sources of nicotine pouches were brick‐and‐mortar stores and “*online*”. In terms of the former, participants reported that nicotine pouches were available from the same stores that sell illegal vapes, with convenience stores and tobacconists (i.e., retail stores that primarily sell tobacco‐related products) specifically mentioned. In terms of online retailers, notable sources mentioned by a few participants included Facebook Marketplace, Telegram, and Discord. The following quotes from women aged 25–39 years in Perth identify various product sources:

Interviewee 1: *They're very easy to get online through customs… Pretty much most places that you can get a vape now, you can get them coming into it. But a lot come through online.* [*unrelated discussion*]

Interviewee 2: *But you can get them anywhere you can get vapes*, *they reckon*.

### 3.4 Retailer Practices

Few retailer practices were identified, with these broadly relating to product cost and inducements. Participants typically commented that nicotine pouches were “*cheap*” or “*cheaper than vapes*”, with few believing them to be more expensive. Some participants also reported that purchasing in bulk from an online source typically attracted a discount:

*I think they're still cheaper than vapes. It depends if you buy them online in bulk* [Men, 25–39 years, Melbourne].

In terms of inducements, some participants commented on the accrual of points when purchasing ZYN. Participants reported that these points can subsequently be redeemed for rewards in the United States:

*Yeah, and you can get rewards. If you have so many boxes, you can get reward points. I think it's only America though, but some people got barbecues and stuff from getting lots of ZYN boxes, and you get a barbecue or something* [Women, 16–17 years, Perth].

### 3.5 Industry Innovation

Some industry innovations were identified, with these primarily related to product packaging. Participants commented on slim container designs, which allowed for more discreet use. They also commented on the presence of a storage compartment for used pouches, which one participant described as a “*smart feature*”:

Interviewee 1: *Also, it has a smart feature. Apparently, it has—you can put the used one there if you don't have a bin or something*.

Interviewer: *In the tin?*

Interviewee 1: *Yeah, it's a section that you can put it in or something* [Women, 16–17 years, Perth].

## 4 Discussion

In light of rapidly increasing sales of nicotine pouches, we sought to explore younger people's exposure to nicotine pouch advertising, from where or whom nicotine pouches are being sourced, and notable retailer practices and industry innovations. Consistent with prior work identifying the “aggressive promotion” of recently developed tobacco and nicotine products [21], almost all groups reported being exposed to pouch advertising, despite this being illegal in Australia [11]. This advertising was not always obvious, with participants discussing the subtle ways in which influencers were promoting pouch use on social media and podcasts. Consistent with research conducted in the UK [13], participants also reported seeing pouches being promoted by athletes, such as international soccer/football players. Men were more likely than women to report being exposed to advertising, suggesting this population cohort may be a target of promotional activities. Indeed, in focus groups conducted with 14 to 16‐year‐olds in the UK, participants held the view that pouch marketing was aimed more at males than females [13]. This targeted advertising appears to be effective, with research indicating that nicotine pouch use is greater among men [5, 6].

Although we did not seek to explore the nature of nicotine pouch advertising, some participants spontaneously disclosed their observations, noting that such advertising typically featured attractive and wealthy influencers portraying the products as “cool”. These aspiration‐based themes are highly reminiscent of tobacco and vape advertising [22, 23, 24], highlighting continued attempts by the industry to associate their products with consumer lifestyles [25]. Participants' observations that fitness/wellness influencers are presenting nicotine as a natural product that is not inherently harmful are of further concern. This narrative may be part of a broader strategy by tobacco and nicotine companies to market their products as clean, performance‐enhancing, and health‐promoting [26]. The promotion of this narrative to young people on social media is concerning given (1) nicotine is highly addictive and (2) nicotine pouches have been found to contain many other chemicals, some of which are carcinogenic [27, 28].

Consistent with prior quantitative work [8], participants commented on the availability of nicotine pouches from brick‐and‐mortar convenience stores and tobacconists, despite the retail sale of the products being illegal in Australia. Participants also reported being able to purchase nicotine pouches online, with social media platforms (e.g., Facebook Marketplace) and communication platforms (e.g., Discord and Telegram) providing a means by which young people could purchase the products from others. Finally, in terms of retailer practices and industry innovations, few were identified. This likely reflects the nature of our sample, with only 16 participants reporting that they had ever used nicotine pouches. As such, most participants had little experience purchasing the products and were therefore unlikely to be able to identify such practices. The practices identified, however, are noteworthy. The products were typically described as cheap or cheaper than vapes, especially when purchased online in bulk (which attracted discounts). These findings mirror observations made in the context of illicit vapes, with the Australian market flooded with cheap, addictive products targeting young people [29].

### 4.1 Implications

While further research on the nicotine pouch market in Australia is warranted, our results provide initial insights into the insidious nature of the marketing strategies being adopted to sell nicotine pouches to younger people and the type of content to which younger Australians are being exposed. Our findings highlight the need for urgent action that protects younger people from the potential harms caused by (1) social influencers who promote addictive products and (2) tobacco and nicotine companies that engage influencers to promote pouch use. This action should include robust regulation of the online environment and social influencers, with social media platforms held accountable for their failure to enforce prohibitions on product advertising and sales. Consistent with Article 13 of the WHO Framework Convention on Tobacco Control [30], greater attention should also be paid to sports sponsorship, with arrangements between athletes, Formula 1 racing teams, and tobacco and nicotine companies prohibited.

In addition to advertising regulation, our research demonstrates that greater action is required to dismantle the illicit trade of nicotine products in Australia. While enhanced border control measures are necessary, greater enforcement and more effective deterrents at the local level are urgently needed. Some Australian states (e.g., Queensland, South Australia) are performing better than others (e.g., Victoria) in this regard, introducing legislation that allows for the immediate closure of retailers found to be engaging in illegal activity.

### 4.2 Limitations

There are two primary limitations to this study. First, our qualitative approach to data collection limits our ability to generalise findings to the broader population of younger Australians. Second, few participants reported engaging in nicotine pouch use, which likely limited our ability to capture detailed information on retailer practices. Further research exploring the experiences of those who use the products is warranted.

## 5 Conclusion

Our research exploring the nicotine pouch market in Australia from the perspective of younger people suggests the products are being primarily advertised by influencers on social media, with aspirational and health‐promoting themes observed. The products are being sourced from convenience stores and tobacconists, as well as online communication platforms. Findings highlight an urgent need for action that protects younger people from the activities of tobacco and nicotine companies and the influencers they employ to promote their addictive products.

## Funding

This work was supported by a National Health and Medical Research Council Investigator Grant (APP1194713) awarded to Professor Michelle Jongenelis and the Victorian Health Promotion Foundation. Funding sources had no involvement in study design; in the analysis and interpretation of data; in the writing of the article; and in the decision to submit the article for publication. Representatives from the Victorian Health Promotion Foundation were present during data collection.

## Conflicts of Interest

The authors declare no conflicts of interest.

## Data Availability

The data that support the findings of this study are available on request from the corresponding author. The data are not publicly available due to privacy or ethical restrictions.
